# Construction of *Aspergillus niger* integrated with cellulase gene from *Ampullaria gigas* Spix for improved enzyme production and saccharification of alkaline-pretreated rice straw

**DOI:** 10.1007/s13205-016-0545-0

**Published:** 2016-11-04

**Authors:** Peizhou Yang, Haifeng Zhang, Lili Cao, Zhi Zheng, Shaotong Jiang

**Affiliations:** 1 College of Food Science and Engineering, Hefei University of Technology, Hefei, 230009 China; 2 Anhui Key Laboratory of Intensive Processing of Agricultural Products, Hefei University of Technology, Hefei, 230009 China

**Keywords:** *Aspergillus niger*, Cellulase gene, In situ saccharification, Alkaline pretreatment, *Ampullaria gigas* Spix

## Abstract

*Aspergillus niger* is an important microorganism that has been used for decades to produce extracellular enzymes. In this study, a novel *Aspergillus niger* strain integrated with a eukaryotic expression vector harboring the gpd-Shi promoter of shiitake mushrooms and cellulase gene of *Ampullaria gigas* Spix was engineered to improve cellulase production for the achievement of highly efficient saccharification of agricultural residues. In one strain, designated ACShi27, which exhibited the highest total cellulase expression, total cellulase, endoglucanase, exoglucanase, and xylanase expression levels were 1.73, 16.23, 17.73, and 150.83 U ml^−1^, respectively; these values were 14.5, 22.3, 24.6, and 17.3% higher than those of the wild-type *Aspergillus niger* M85 using wheat bran as an induction substrate. Production of cellulases and xylanase by solid-state fermentation followed by in situ saccharification of ACShi27 was investigated with alkaline-pretreated rice straw as a substrate. After 2 days of enzyme induction at 30 °C, followed by 48 h of saccharification at 50 °C, the conversion rate of carbon polymers into reducing sugar reached 293.2 mg g^−1^, which was 1.23-fold higher than that of the wild-type strain. The expression of *sestc* in *Aspergillus niger* can improve the total cellulase and xylanase activity and synergism, thereby enhancing the lignocellulose in situ saccharification.

## Introduction

In recent years, as the consumption of crude oil has increased, the development of renewable biofuels has become an important focus of many researchers; in particular, the potential applications of lignocellulose ethanol have been recognized (Kuhad et al. 2016). Lignocellulosic ethanol is advantageous over other potential biofuels owing to its capacity for the reduction of greenhouse gas emissions and lower cost of raw materials.

Conversion of lignocellulose into ethanol includes three essential steps: pretreatment, saccharification, and ethanol production (Limayem and Ricke 2012). During cellulose saccharification, cellulose hydrolysis can be accomplished using acid treatment; however, saccharification is most efficient when using cellulase and xylanase enzymes (Sukumaran et al. 2009). Such enzymatic degradation is thought to be more environmental friendly and produces fewer inhibitors, which suppress the subsequent conversion, further improving the efficiency of the reaction(Rosgaard et al. 2007). However, the high cost of cellulase has limited its applications in the bioenergy industry (Favaro et al. 2013; Klein-Marcuschamer et al. 2012). Multifaceted approaches, including the utilization of inexpensive raw materials, such as agricultural waste, and more efficient strategies for enzyme production, such as solid-state fermentation, could reduce the production cost of cellulase (Behera and Ray 2016; Moshi et al. 2015; Yoon et al. 2014). Improving cellulase production by microbial strains is also a promising approach to achieve these goals (Garvey et al. 2013). Multicomponent enzyme systems primarily contain endoglucanases [endo-1,4-β-glucanases (EGs); EC 3.2.1.4], exoglucanases [exo-1,4-β-glucanases (CBHs); EC 3.2.1.91], and β-glucosidases [β-d-glucohydrolases (BGLs); EC 3.2.1.21] (Garvey et al. 2013). EGs and CBHs synergistically convert cellulose chains into cellobiose and other oligosaccharides, which are then hydrolyzed by β-glucosidases to liberate glucose (Bansal et al. 2012).

Several microorganisms, including those from the genera *Bacillus*, *Clostridium*, *Cellulomonas*, *Aspergillus*, and *Rhizopus*, produce cellolytic enzymes (Vijayaraghavan et al. 2016). *Aspergillus niger* has many characteristics that are advantageous for industrial applications, such as superior fermentation capabilities and high levels of protein secretion (de Vries and Visser 2001). The BGLs from *A. niger* exhibit higher specific activities than those of other fungi (Dotsenko et al. 2015). However, EG and CBH are not highly involved in the cellulase system of *A. niger* (Yoon et al. 2014). Importantly, *A. niger* can produce other accessory enzymes of biomass degradation, such as xylanases, xyloglucanases, and α-l-arabinofuranosidases (Gusakov 2011). As the progress in heterologous expression, production systems of recombinant enzyme have proved to be promising platforms to efficiently produce industrial cellulase (Garvey et al. 2013).

Increasing the performance of CBH and EG in the enzyme system of *A. niger* may improve the activity of the cellulase system. Accordingly, in this study, a strain of *A. niger* harboring the *sestc* gene was engineered under control of the *gpd* promoter to improve cellulase activity. The *sestc* gene encodes a protein containing CBH, EG, and xylanase activities and has been shown to be effectively expressed in many other hosts (Cheng et al. 2009; Wang et al. 2003). We expected that expression of the *sestc* gene could ultimately enhance degradation of lignocellulose-based biomass. Thus, using the engineered strain, we evaluated the production of the reducing sugar released from pretreated rice straw by solid-state fermentation and in situ saccharification.

## Materials and methods

### Plasmid and strains

The plasmid pBluescriptIIKS was used as the backbone for the eukaryotic expression vector. The *gpd*-*Shi* promoter from shiitake mushrooms and the single enzyme system triplicate cellulase activity (*sestc*) gene from *Ampullaria gigas* Spix were fused and inserted as described previously (Okada et al. 1998; Watanabe et al. 2016). The hygromycin B phosphotransferase gene (*hph*) served as the selection maker, and the length of the entire plasmid (pgShi-sestc-hph) was 6380 bp. The primers used in this study are listed in Table 1. The host strain *Aspergillus niger* M85 was obtained from China Center of Industrial Culture Collection, preserved in the Experimental Center of College of Food Science and Engineering. The cell wall lytic enzyme was from Guangdong Institute of Microbiology.

**Table 1:** Primers used for PCR amplification

Primer name	Fragment	Sequence (5′–3′)
gpdShi_F	*gpd* promoter fragment	ATTCAAGCAGTCAATGGATTGG
gpdShi_R	*gpd* promoter fragment	GAAGTTTGAGGTGGTTGCGAATAC
sestc_F	*sestc* gene ORF fragment	TTCGTCGACGCTTC
sestc_R	*sestc* gene ORF fragment	GATTATGCCCTCTGAGTGTC
gpd-sestc_F	gpd-sestc with *Sac* II and *BsrG* I	ggccgcggATTCAAGCAGTCAATGGATTGG
gpd-sestc_R	gpd-sestc with *Sac* II and *BsrG* I	ccggtgtacacGATTATGCCCTCTGAGTGTC
sestc600_F	*sestc* gene fragments	GTCGGCGGCGTGTGCGATACG
sestc600_R	*sestc* gene fragments	GCTTCAGTCAAGCGCATGCC

F and R referred to forward and reverse primers, respectively; bases for restriction sites and protection bases were shown in lowercase letters; *Sac* II and *BsrG* I restriction sites are *underlined*

### Protoplast preparation

The wild-type *Aspergillus niger* M85 strain was conserved in potato dextrose agar slants at 4 °C and subcultured monthly. The expression plasmid was transferred into *A. niger* using the protoplast method. *Aspergillus niger* M85 from PDA slants was inoculated into agar slants of wheat bran prepared with 50 g wheat bran, 10 g (NH_4_)_2_-HPO_4_, 0.2 g K_2_HPO_4_, 0.1 g MgSO_4_·7H_2_O, 20 g agar, and 1 L distilled water (pH 7.0). The spores were accumulated with stationary culture for 7 days at 30 °C. Then, the slant pores were washed using 2 ml sterile water to suspend spores with a concentration of 10^8^ ml^−1^. A spore suspension of 1 ml was added to a 250-ml Erlenmeyer flask containing 100 ml YPD medium, prepared with 1% yeast extract, 2% peptone, and 2% glucose. The suspension was then incubated for 48 h at 30 °C with shaking at 180 rpm. The mycelia were collected by filtration through two layers of gauze and ground in a sterile mortar and pestle. A fungal paste of 0.5 g was suspended in 5 ml of 2% lywallzyme solution, prepared with 0.1 g lywallzyme and 5 ml sorbitol (1 M) and sterilized by filtration. After digestion for 1 h at 30 °C and centrifugation at 80 rpm, mycelial debris was removed physically filtered through three layers of lens paper. The filtrate was harvested by centrifugation at 3200 rpm for 10 min, and the supernatant was then gently discarded. The protoplasts were washed with ice-cold 0.6 M KCl and S/C, prepared with 1 M sorbitol and 50 mM CaCl-_2_. The protoplasts were then resuspended in an S/C solution of 1.5 ml at a concentration of about 1–3 × 10^6^ ml^−1^.

### Transformation, screening, and molecular identification

Plasmid DNA (1 μg) was added to a protoplast suspension of 100 μl and then mixed for 20 min on ice with 50 μl polyethylene glycol (PEG) buffer, prepared with 25% PEG8000, 50 mM CaCl_2_, and 10 mM Tris–HCl adjusted to pH 7.5. After incubation at room temperature for 5 min, the reaction system was mixed with 2 ml S/C solution. This solution was then diluted tenfold using S/C solution. One hundred microliters of the diluted solution was then evenly coated on the regeneration agar medium, prepared with 0.3% yeast extract, 1% peptone, 0.6 M MgSO_4_, 2% glucose, 1.8% agar, and 120 μg ml^−1^ hygromycin B, and the samples were cultivated for 72 h at 30 °C (Kusters-van Someren et al. 1991; Meyer et al. 2003). The regeneration colonies were picked out and identified using polymerase chain reaction (PCR) (Karakousis et al. 2006). For the putative transformants, the total RNA was extracted after cultivation for 72 h in YPC medium containing 10 g l^−1^ yeast extract, 20 g l^−1^ peptone, and 5 g l^−1^ CMC-Na. Mycelial pellets were collected, frozen in liquid nitrogen, and ground for 1 min to a fine powder (Vries et al. 1989). Total RNA was purified using omega Total RNA kit according to the manufacturer’s protocol (Omega, USA), and the RT-PCR was performed using a PrimeScript RT-PCR Kit (TaKaRa, Shiga, Japan). cDNA encoding *sestc* was amplified by PCR with synthetic primers.

### Clearance zone identification

To evaluate enzyme hydrolysis activity, the transformants cultivated for 36 h on YPD agar plates were spot plated on carboxy methylcellulose (CMC) agar medium containing 0.2% NaNO_3_, 0.1% K_2_HPO_4_, 0.05% MgSO_4_, 0.05% KCl, 0.2% CMC sodium salt, 0.02% peptone, and 1.7% agar and cultivated for 72 h at 30 °C. Gram’s iodine solution (30 ml), prepared using 1 g iodine and 2 g KI in 300 ml distilled water, was flooded on the CMC agar plate for 5 min. The sizes of clearance zones were determined using Vernier calipers based on the diameter of the outer circle (Peizhou et al. 2016).

### Cellulase expression under submerged fermentation

The wild-type *Aspergillus niger* M85 strain and three recombinant *A. niger* strains were cultivated in 250-ml Erlenmeyer flasks containing 100 ml induction fermentation medium, containing 1 g l^−1^ peptone, 0.3 g l^−1^ urea, 2 g l^−1^ KH_2_PO_4_, 0.2 g l^−1^ CaCl_2_, 0.3 g l^−1^ MgSO_4_·7H_2_O, 5 mg l^−1^ FeSO_4_·7H_2_O, 1.6 mg l^−1^ MnSO_4_·7H_2_O, 1.4 mg l^−1^ ZnSO_4_·7H_2_O, 20 mg l^−1^ CoCl_2_·6H_2_O, 10% (w/v) wheat bran, and 0.11% (w/v) Tween-80. The wheat bran was from the native cultivated variety in Hefei area, China. The main components of wheat bran were 12.5% starch, 5.5% fat, 18.8% crude protein, 4.5% ash, 16.2% cellulose, 29.4% hemicellulose, 13.1% other compounds. Flasks were shaken on an orbital shaker at 180 rpm and 30 °C. The fermentation broth was withdrawn and centrifuged for 10 min at 4 °C. Supernatants were used to assay the filter paper activity (FPA, total cellulase activity), CMCase (endo-β-1-4-glucanase) activity, cellobiohydrolase (exo-β-1-4-glucanase) activity, and xylanase activity at 50 °C and pH 4.8. The corresponding substrates were Whatman No. 1 filter paper (50 mg, 1 × 6 cm), 0.51% (w/v) CMC, 1% (w/v) microcrystalline cellulose, and 1% (w/v) xylan, respectively. The substrates were dissolved in 0.05 M sodium citrate buffer (pH 4.8). One unit of enzymatic activity was defined as the amount of enzyme producing 1 μmol/min reducing sugar at 50 °C.

### Pretreatment and in situ rice straw saccharification

The rice straw was harvested from a local farm, ground, and sieved to 3-mm size after drying in a drying oven at 60 °C for 36 h. The milled biomass was slurried in 1 N NaOH with a ratio of 1:10 (w/v) and autoclaved at 121 °C and 15 psi for 1 h. After washing with distilled water to pH 7.2, filtering and squeezing desalted biomass using gauze to remove the water, then drying in a drying oven at 60 °C for 48 h, the alkali-free pretreated rice straw was used to prepare fermentation medium to induce the expression of components of the lignocellulase system. The different components of rice straw were measured as previously described (Sridevi et al. 2015). The solid-state fermentation was carried out in 100-ml Erlenmeyer flasks containing 5 g pretreated dried rice straw and moistened with 15 ml Mandels mineral medium (containing 0.11% Tween-80). The flasks were autoclaved at 121 °C for 20 min, followed by inoculated with 1-ml spore suspensions of strain ACShi27 and incubated at 30 °C. As the control, the same amount of *Aspergillus niger* M85 spore suspension was inoculated into the fermentation medium, and same flasks not inoculated spore served as blank control group During the period of cultivation, the cellulase and xylanase activities were measured daily. The enzyme activity was expressed in terms of unit per gram of solid substrate (U g^−1^). After enzyme induction, The fungus and medium were submerged in 50 ml of 50 mM citrate buffer (pH 4.8) and incubated in a water bath at 50 °C for 48 h. Samples were harvested by filtration and centrifugation for reducing sugar analysis using the dinitrosalicylic acid (DNS) method (Miller 1959).

### Statistical analyses

The statistical tool SPSS was employed for statistical analysis. All fermentations and assays were performed in triplicate, and *p* values of less than 0. 1 were considered significantly different. Error bars were determined from triplicate samples based on the standard deviation from the mean values.

## Results and discussion

### Process of obtaining positive transformed clones

#### Construction of an expression vector integrated with *sestc* and *hph* cassette

**Fig. 1:**
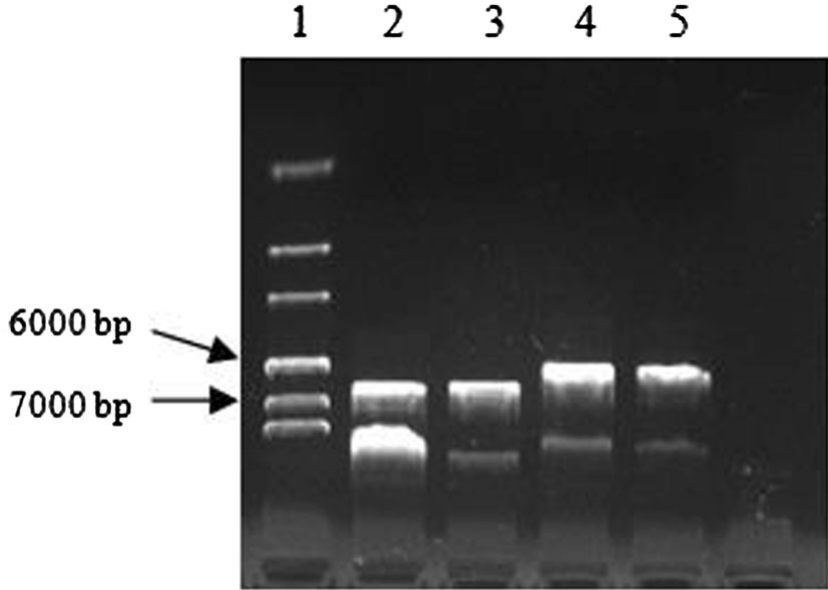
Construction of the expression vector pgShi-sestc-hph. *Lane 1* marker; *lanes 2*, *3* pgShi-sestc-hph; *lanes 4*, *5* pgShi-sestc

**Fig. 2:**
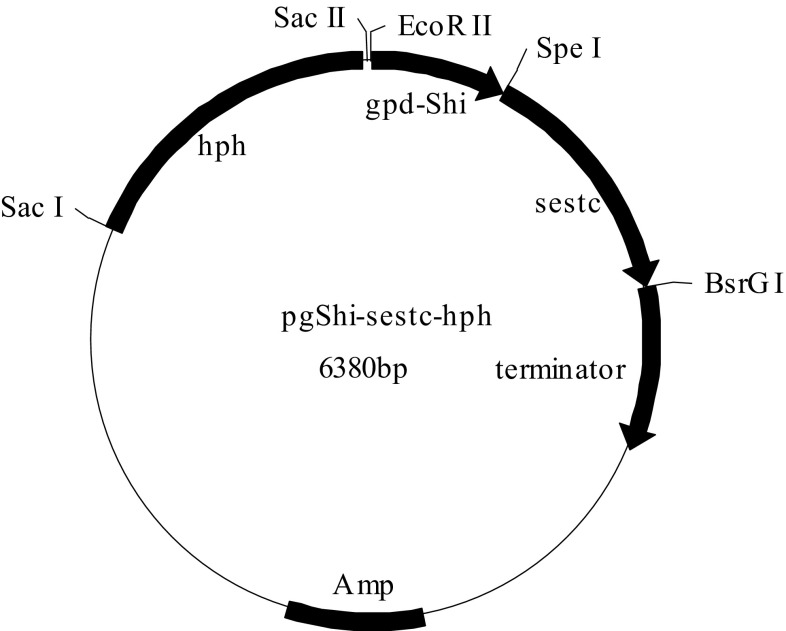
Schematic representation of the structure of the expression vector pgShi-sestc-hph

The DNA genome of shiitake mushrooms was amplified using the designed primers gpdShi_F and gpdShi_R to isolate the glyceraldehyde-3-phosphate dehydrogenase (*gpd*) promoter (*gpd*-*Shi*). Primers sestc_F and sestc_R were used to amplify the cDNA of *Ampullaria gigas* Spix to isolate the *sestc* gene. Primers gpd-sestc_F and gpd-sestc_R were applied to facilitate the integration of *gpd*-*Shi* and *sestc* into the framework of pBluescriptIIKS. The new expression vector was named *pgShi*-*sestc* (Fig. 1). The *hph* expression cassette and vector *pgShi*-*sestc* were then assembled by molecular methods to produce the expression vector *pgShi*-*sestc*-*hph* (Fig. 1). The size of the expression vector *pgShi*-*sestc*-*hph* was 6380 bp (Fig. 2). All the PCR products were inserted into pGEM ^®^vectors. And the amplified fragments were confirmed to be correct as expected with DNA sequencing by Shanghai Sangon Biotech Company (China).

#### Protoplast preparation

Following treatment with lywallzyme, the outer cell wall of *A. niger* was degraded, and the membranes were exposed. In the outer solution, the osmotic pressure formed from the dissolved sorbitol and CaCl_2_ was similar to that inside the protoplast cells, ensuring that the cells remained intact. After incubation in a warm bath for 1 h, a protoplast content of 84% was achieved.

Prolonging the treatment time increases the content of *A. niger* protoplasts. However, excessive enzymatic hydrolysis decreases transformation efficiency (Dawe et al. 2000). Protoplasts are an efficient biological tool that can be used for molecular genetics transformation. Many applications are based on the assumption that the protoplasts are physiologically normal and maintain the biological properties of intact cells. The lywallzyme cocktail contains lysing enzymes, chitinase, and β-glucuronidase (de Bekker et al. 2009).

#### Determination of the minimum hygromycin B concentration

*Aspergillus niger* protoplasts were prepared at a concentration of 10^8^ ml^−1^ to investigate the effects of hygromycin B concentration on protoplast regeneration. A protoplast solution (150 μl) was coated on PDA medium containing hygromycin B. Our analysis demonstrated that the minimum concentration of hygromycin B was 120 μg ml^−1.^

Resistance screening of genetic transformation is an important method required to obtain true transformants. In this study, *Aspergillus nidulans gpd* and *trpC* expression signals controlled the expression of the *hph* gene. *Aspergillus* species cannot grow on solid plates containing certain concentrations of hygromycin B. Heterologous expression of *hph* from *Escherichia coli* in the *A. niger* can alleviate resistance inhibition (Punt et al. 1987).

#### Transformation and identification by PCR and RT-PCR

**Fig. 3:**
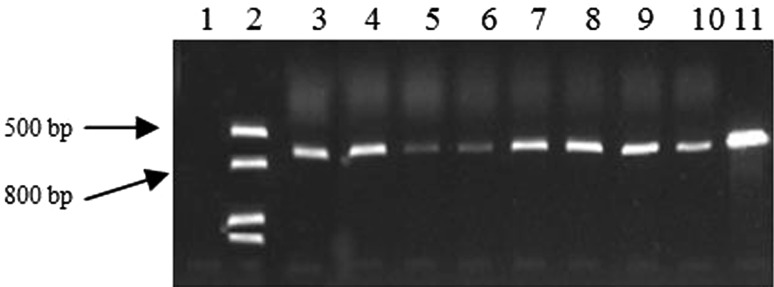
PCR amplification of *Aspergillus niger* transformants selected according to hygromycin B resistance. *Lane 1* wild-type control; *lane 2* marker; *lanes 3*–*10 A. niger* transformants; *lane 11* positive control

After screening of plates supplemented with 120 μg ml^−1^ hygromycin B, 96 *A. niger* were selected for PCR identification. Bright bands from the genomic DNA of 85 colonies were amplified, showing sizes similar to that of the positive control (Fig. 3).

**Fig. 4:**
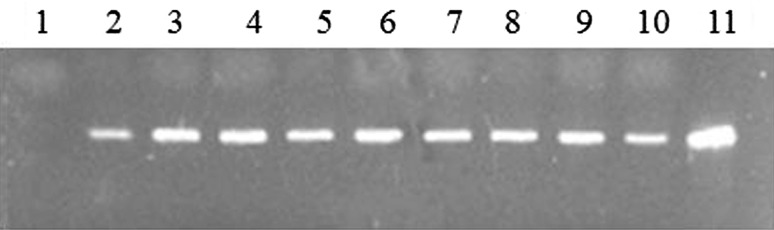
Identification of transformants using RT-PCR. *Lane 1* wild-type *Aspergillus niger*; *lanes 2*–*10 A. niger* transformants; *lane 11* positive control. PCR product identification was further confirmed using gene sequencing method

Based on the PCR identification, the 85 positive transformants were further investigated using RT-PCR analysis. Using the primers sestc600_F and sestc600_R to amplify a PCR product of 600-bp size, we found that 73 of the 85 transformants were positive (Fig. 4). Thus, the overall transformation rate was 76% based on the original 96 hypothetical transformants. Thus, in these positive transformants, the *sestc* gene had inserted into the chromosomal DNA of *A. niger*, and *sestc* mRNA was effectively expressed at high levels under control of the *gpd*-*Shi* promoter.

The DNA band brightness of amplification products was observed by electrophoresis gel imaging system. The comparison of cellulase expression among those identified transformants was investigated with wheat bran as fermentation substrate. Based on a combination of high cellulase activity and strong brightness bands, three transformants named ACShi16, ACShi20, and ACShi27 were chose for further analysis.

### Clear zone assays

**Fig. 5:**
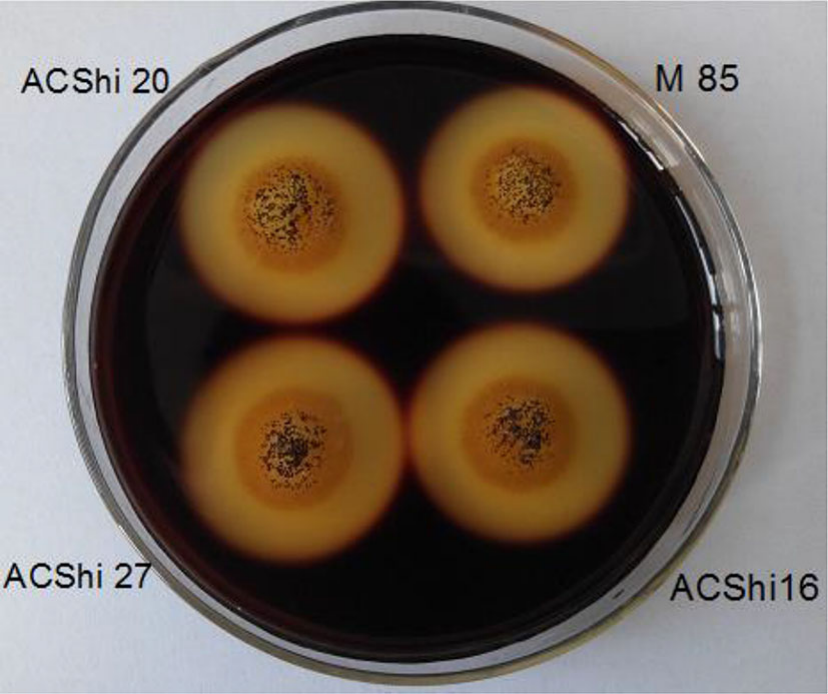
Clear zone determination for wild-type *Aspergillus niger* M 85 and the engineered *A. niger* strains

The wild-type *Aspergillus niger* M85 and three transformants were spot plated on CMC agar plates and cultivated for 3 days. After staining with Gram’s iodine solution, transparent circles were visible; the diameters of these circles were correlated with the secretion of cellulase. The outer layer diameter of strain M85 was 2.76 ± 0.05 cm, whereas those of ACShi16, ACShi20, and ACShi27 were 2.90 ± 0.08, 2.94 ± 0.11, and 3.11 ± 0.08 cm, respectively (Fig. 5). The cellulase activities of the transformants were higher than that of the wild-type strain. Among the identified transformants, the largest circle diameter, observed for *Aspergillus niger* ACShi27, was 12% larger than that of the wild-type strain.

### Cellulase expression under submerged fermentation

**Fig. 6:**
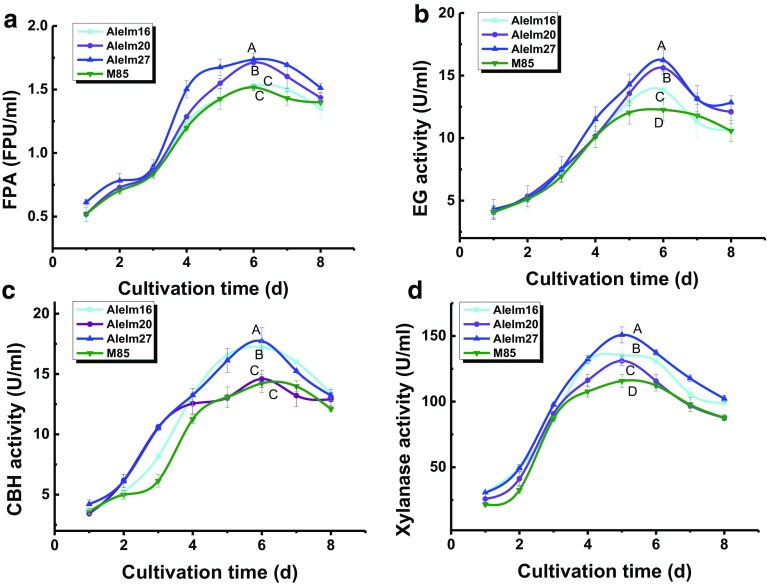
Enzymatic determination of total cellulase (**a**), EG (**b**), CBH (**c**), and xylanase (**d**) activities in *Aspergillus niger* M85, AShim16, AShim20, and ACShi27 by submerged fermentation. The significant differences of each enzyme activity (Turkey’s test, *p* < 0.1) were represented by different characters (A, B, C, D). *Error bars* were determined from triplicate samples based on the standard deviation from the mean values

Previous studies have shown that wheat bran is an efficient lignocellulase inducer (Sukumaran et al. 2009; Camassola and Dillon 2007; Sharma et al. 2015). In the present study, the wild-type strain was used as the control, three transformants (ACShi16, ACShi20, and ACShi27) were inoculated in liquid medium containing 10% wheat bran. The fermentation broths from three transformants and the wild-type strain were harvested daily. The activities of total cellulase, EG, CBH, and xylanase from the four strains are shown in Fig. 6. The highest activity levels appeared after incubation for 6 days. Additionally, the highest xylanase activity was observed after fermentation for 5 days. All enzyme activities from the three transformants were higher than those of the wild-type M85 strain. The highest enzyme activities were observed in the ACShi27 strain, in which total cellulase, EG, CBH, and xylanase activities were 1.73 ± 0.05, 16.23 ± 0.92, 17.73 ± 1.11, and 150.83 ± 6.05 U ml^−1^, respectively; these values were 14.5, 22.3, 24.6, and 17.3% higher than those of the wild-type M85 strain (Fig. 6). The native *A. niger* strain exhibits low EG and CBH activities (Wang et al. 2015), leading to an imbalance in the enzyme components of the lignocellulytic hydrolysis system. In this study, we used the protein coding gene *sestc* from *Ampullaria gigas* Spix, which exhibits EG, CBH, and endo-β-1,4-xylanase activities (Wang et al. 2003), providing activities to complement those of the lignocellulytic enzyme system of *A. niger*. The differences in enzyme production among the three engineered strains could be explained by different gene copy numbers and insertion sites.

Plant cell walls contain a complex system composed of 15–40% cellulose, 10–30% hemicellulose, and 5–20% lignin (Bhattacharya et al. 2015). This complex chemical composition and associated recalcitrant physical features can affect the saccharification process. Enhancing the overall effects of natural biomass degradation requires the efficient synergism of cellulases and hemicellulases. Reported lignocellulytic enzymes in native *A. niger* include endoglucanase (Egl C, GH74) (Hasper et al. 2002), endo-β-1-4 xylanse (Xln A, GH 10) (Do et al. 2013), endoglucanase (EglA, GH 12) (Pham and Nghiem 2011), xyloglucanase (AnXEGl2A, GH 12) (Master et al. 2008), and α-l-rhamnosidase (GH 13) (Liu et al. 2012), among others. However, wild-type *A. niger* generally expresses and secretes EGs at low levels (Busto et al. 1996), suggesting that there may be an imbalance in the expression of lignocellulolytic hydrolysis enzymes during induction by cellulose substrates. In a single microorganism, maintaining synergistic expression of enzymatic components involved in lignocellulytic hydrolysis is difficult during induction with cellulosic substrates. Although substrate type and cellulase component are known to affect the degree of synergism, the mechanisms mediating this synergism between complex lignocellulose components is not yet completely understood. However, there is a clear relationship between EG and cellobiohydrolase, which function synergistically to hydrolyze the biomass feedstock (Woodward 1991). Moreover, in this study, we found that integration of the single *sestc* gene encoding a multi-functional cellulase overcame the limitations of synergetic expression.

### In situ saccharification of pretreated rice straw

After dilute alkaline pretreatment, the contents of hemicelluloses, lignin, and cellulose used in this study were 24.4% ± 0.29%, 3.7% ± 0.08%, and 61.3% ± 0.70%, respectively (Table 2). Recently, several novel techniques were developed to transform biomass to sugars. Combination of wet oxidation, steam explosion and enzymatic hydrolysis was used to produce sugars (Rana et al. 2012). A combination of two fungi *Trichoderma reesei* RUT-C30 and *Aspergillus saccharolyticus* could efficiently produce cellulase to degrade pretreated corn stover (Rana et al. 2014). However, under limited conditions mild alkaline pretreatment methods still are considered an efficient technique for destroying the recalcitrant structure of agricultural residues (Yoon et al. 2013). Lignin is thought to be the main contributor to lignocellulolytic recalcitrance, and removal of lignin could markedly promote the accessibility and digestibility of such residues (Carvalho et al. 2015). Alkaline pretreatments cause delignification, disruption of structure linkages, decrystallization of cellulose, and depolymerization of carbohydrates. Moreover, most lignin molecules and partial hemicelluloses are dissolved in alkaline solution.

**Table 2:** Constituent contents for native and dilute alkaline-pretreated rice straw as a percentage based on dry weight

Constituents	Value (%)	
	Native rice straw	Pretreated
Cellulose	49.1 ± 0.41	61.3 ± 0.70
Hemicellulose	30.2 ± 0.39	24.4 ± 0.29
Lignin Others	6.3 ± 0.09 14.2 ± 0.19	3.7 ± 0.08 10.4 ± 0.21

Values were the averages ± standard deviation of three independent experiments

**Fig. 7:**
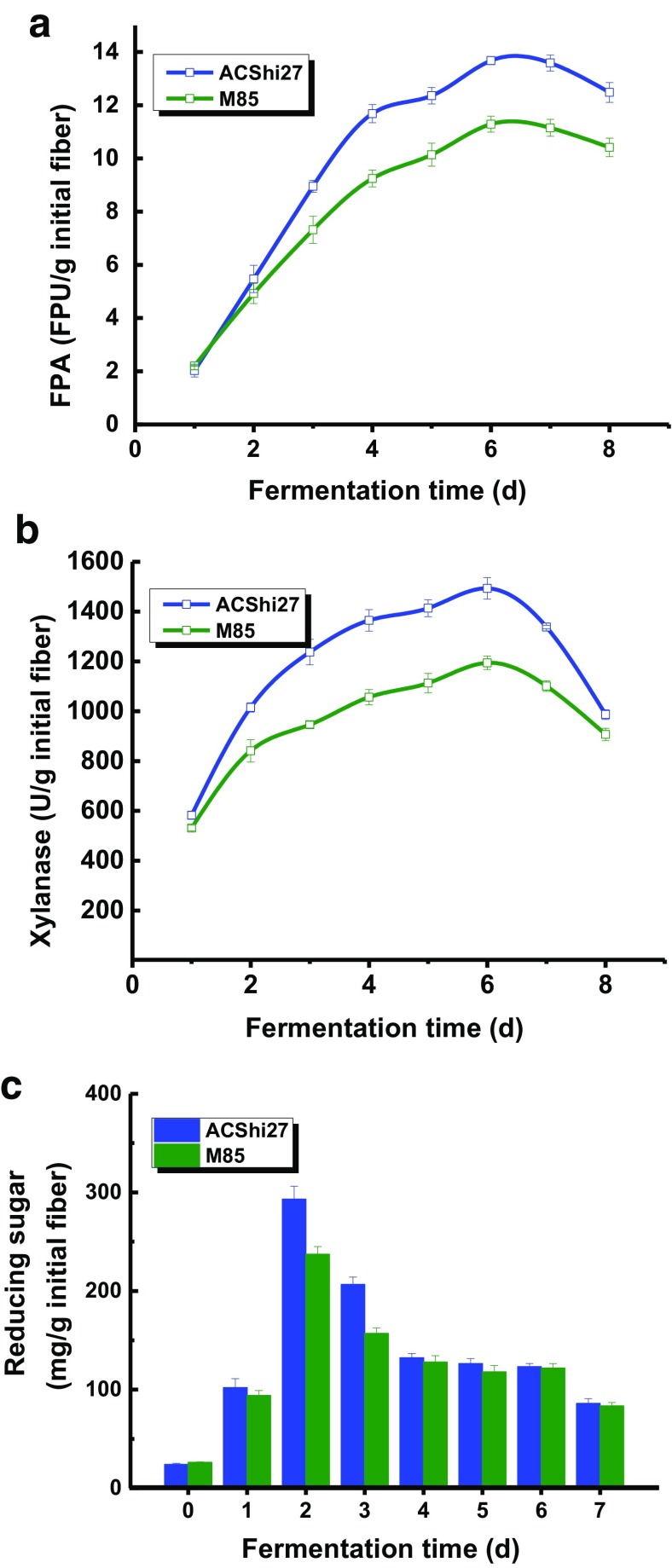
Temporal profiles of FPase activity (**a**) and xylanase activity (**b**) during solid-state fermentation (carbon source: alkaline-pretreated rice straw), as well as reducing sugar released for 48 h (**c**) in ACShi27 and M85. *Error bars* were determined from triplicate samples based on the standard deviation from the mean values

To decrease the cost of cellulase for saccharification, enzymatic hydrolysis in situ was employed to investigate the application value of the engineering *A. niger*. In situ saccharification require for not only efficient lignocellulytic enzyme production but also synergism of enzyme cocktails. Cellulase and xylanase production were carried out at 30 °C, followed by hydrolysis at 50 °C. With a solid-state fermentation technique, the engineered *A. niger* ACShi27 strain was used to produce cellulases with alkaline-pretreated rice straw as an induction substrate. The highest total cellulase and xylanase reached 13.67 and 1493.1 U g^−1^ after fermentation at 30 °C for 6 days; these values were 1.21-fold and 1.25-fold higher than those of the wild-type strain M85 (Fig. 7a, b).

During the solid-state fermentation process, although the saccharifying enzyme activity in medium is sufficient, the reduced sugar did not accumulate because the reduced sugar released by lignocellulose degradation is consumed by the fungus (Rasmussen et al. 2010). Therefore, we performed another set of experiments in which the samples were fermented for 1–7 days, followed by immersion in 50 ml buffer (pH 4.8) to block the consumption of the resulting reducing sugars and incubation in a water bath at 50 °C for 48 h. The reducing sugar content is presented in Fig. 7c. The highest conversion rate of carbon substrate was 293.2 mg g^−1^ after enzymatic induction for 2 days; this was 1.23-fold higher than that of the wild-type strain. Additionally, after 2 days of solid-state fermentation, 5.47 FPU g^−1^ cellulase activity, and 1015.2 U g^−1^ xylanase activity were produced; these values were 1.11- and 1.21-fold higher than those of the wild-type strain, respectively, and this high saccharification rate of substrate which was disproportionate to enzyme activity between ACShi27 and wild-type could be attributed to the synergism of the ACShi27 enzyme system. The conversion rate was 126.3 mg g^−1^ after fermentation for 6 days, representing the highest cellulase and xylanase activities achieved in this study. The imbalance between the conversion rate and enzymatic activity could be explained by the gradual consumption of the lignocellulosic biomass by the microorganism during the enzymatic induction period.

The conversion rate of biomass and sugar was one of the most important indexes for cellulase engineered strain construction and biomass utilization. In this study, the conversion rate of carbon polymers into reducing sugar was 293.2 mg g^−1^. The sugar yields reached 55% for wet-exploded corn stover hydrolyzed with cellulase produced by *Reesei* RUT-C30 and *Aspergillus saccharolyticus* (Rana et al. 2014). It seemed that the conversion rate in this study was lower than that of Rana’s report (Rana et al. 2014). In fact, the denominators calculating conversion rate were from fermentation origin substrate for this study and new pretreated corn stover for Rana’s report, respectively. Undoubtedly, the partial straw substrate was consumed during the process producing cellulase, which may be the main reason that calculated conversion rate of this study was relatively low. For in situ cellulase production and saccharification, fungus *Gloeophyllum trabeum* was employed to convert to about 11% of the corn fiber into sugars (Rasmussen et al. 2010). The sugar yield from *Gloeophyllum trabeum* was lower than that of this study. A significant advantage in this study was to effectively achieve one-site cellulase production and biomass hydrolysis.

## Conclusions

The present work showed that the *Ampullaria gigas* Spix cellulase gene *sestc* expressed by *A. niger* dramatically boosted the performance of the lignocellulytic system of this fungus, as demonstrated by suspended-fermentation and in situ saccharification of rice straw.

**Fig. 8:**
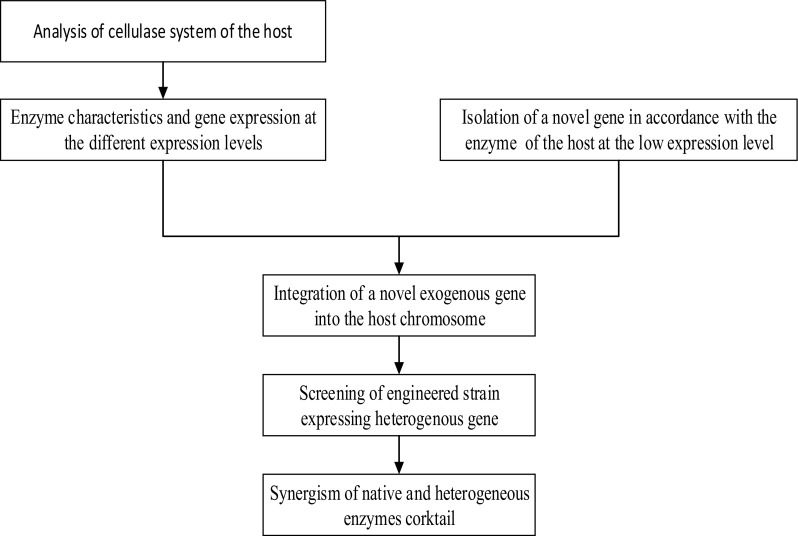
Approach for integration of heterogeneous genes to increase the overall cellulase activities of the engineered strain

The depolymerization of lignocellulosic feedstock using enzyme treatment may contribute to a greener future. Lignocellulytic enzymes may be the key to commercialization of biomass sugar owing to their function in disrupting the complex structure of such sugars. However, several glycosyl hydrolases, including cellulases and hemicellulases, are required for efficient processes. Supplementation with exogenous glycosyl hydrolases promotes the synergistic action of lignocellulytic enzymes in the conversion of polymeric carbons into monosaccharide and oligosaccharides. Accordingly, in this study, we achieved expression of a single cellulase gene, *sestc*, which exhibited high synergy with the native cellulases system. This novel approach is illustrated in (Fig. 8). Our findings demonstrated that this system could be used for more efficient for cellulase activity production and industrialization of biofuel generation.

Notably, during the process of engineering strain construction, the resistance screening marker *hph* was employed to efficiently screen positive transformants; this may potentially threaten environmental security. Future studies are required to investigate more effective screening technologies and identify more efficient promoters.
